# Protective Effects of Propolis and Chitosan Nanoparticles against Ibuprofen-Induced Hepatotoxicity in Albino Rats

**DOI:** 10.3390/diseases12030049

**Published:** 2024-02-29

**Authors:** Fajer M. AlKandari, Hussein S. Mohamed, Sayed A. Ahmed, Basant Mahmoud, Asmaa M. Mahmoud

**Affiliations:** 1 Biochemistry Department, Faculty of Science, Beni-Suef University, Beni-Suef 62511, Egypt; 2 Chemistry Department of Medicinal and Aromatic Plants, Research Institute of Medicinal and Aromatic Plants (RIMAP), Beni-Suef University, Beni-Suef 62511, Egypt; 3 Chemistry Department, Faculty of Science, Beni-Suef University, Beni-Suef 62511, Egypt

**Keywords:** hepatotoxicity, non-steroid anti-inflammatory drugs, propolis, chitosan nanoparticles, anti-inflammatory, anti-oxidative

## Abstract

Post-marketing hepatotoxicity findings are more common or occur much later. NSAIDs (non-steroidal anti-inflammatory drugs) like ibuprofen are consumed in large quantities around the world. NSAIDs have a low incidence of hepatotoxicity but their wide use makes them a major contributor to drug-induced liver injury. Hepatitis is linked to systemic oxidative stress which results in cellular necrosis and fibrosis, as well as tissue lipoprotein peroxidation and glutathione depletion. Given the lack of safe and effective anti-hepatitis drugs in medicine today, natural substances appear to be a promising and safe alternative. Propolis and chitosan are considered natural substances that have a protective effect on the hepatocytes. The purpose of this study was to validate the protective effect of propolis/chitosan nanoparticle extracts on ibuprofen-induced hepatotoxicity. Thirty (30) albino rats were used for the experiment. Animals were exposed to ibuprofen (400 mg/kg body weight/day) for 4 weeks (7 days/week) followed by treatment with propolis (200 mg/kg body weight/day) and chitosan extract (200 mg/kg body weight/day) separately and also in combination for consecutive 4 weeks. This study revealed a significant increase in serum transaminases, alkaline phosphatase, albumin, and total bilirubin in serum, as well as an increase in lipid peroxidation (MDA) and nitric oxide (NO). Furthermore, GSH, GST, and SOD decreased significantly in the group that was exposed to ibuprofen. Furthermore, there was a significant increase in pro-inflammatory parameters such as IL-1β and NF-ĸB, as well as low levels of anti-inflammatory parameters such as IL-6 and BCl-2. These alterations were improved by propolis and chitosan extracts, which was further confirmed in experimental animals. This study demonstrated that propolis and chitosan nanoparticle extracts have the potential to protect against hepatotoxicity induced by ibuprofen, due to their ability to regulate anti-inflammatory and anti-oxidative defense activities.

## 1. Introduction

Maintaining the internal environment of the body is primarily the function of the liver. At this time, liver function cannot be restored. Basically, it controls the flow of nutrients and how proteins, fats, and carbohydrates are metabolized. Liver damage is largely caused by drug use [1]. Hepatotoxicity is the term used to describe chemical-induced liver injury. Even when administered within therapeutic bounds, several drugs have the potential to cause harm to the organ if overdosed or given infrequently. More than 900 medications have been connected to liver damage, which is the most common reason for drug withdrawals [2].

Non-steroidal anti-inflammatory drugs (NSAIDs), such as acetaminophen, nimesulide, diclofenac, and ibuprofen, are the mainstay of pharmaceutical treatment for the majority of rheumatological disorders. Both on prescription and off-label, they are widely used as analgesics and antipyretics. It is the primary factor in the hazardous drug-induced harm to multiple organ systems, including the well-known renal and gastrointestinal injuries [1].

Medications used to treat musculoskeletal system diseases were the second-most common cause of adverse drug events (ADE), accounting for 14% of all reported ADEs, according to Thai FDA statistics from 1984 to 2016. The top 15 medicines to induce ADE include ibuprofen and diclofenac [3]. Hepatic adverse effects from NSAIDs might vary from fulminant liver failure to asymptomatic elevations in blood aminotransferase levels and hepatitis with jaundice. NSAIDs have been connected to many side effects in addition to their well-documented principal side effect, harm to the gastrointestinal mucosa [4].

The use of certain drugs can cause liver toxicity. Nonsteroidal anti-inflammatory drugs (NSAIDs) play an important role in liver damage [5]. NSAIDs can cause liver harm in more than one way. Hepatic metabolism of NSAIDs leads to injuries including mitochondrial injury, increase in liver enzymes such as aminotransferases, and oxidative stress [6]. Ibuprofen (IBP), an NSAID with antipyretic and analgesic properties, inhibits the cellular enzymes cyclooxygenase-1 and 2 [7]. In addition, IBP inhibits mitochondrial beta-oxidation and ATP production through thioester formation with coenzyme A. Defects in fatty acid beta-oxidation lead to microvascular steatosis, which in severe form can lead to liver failure, coma, and death [8]. These defects arise from the accumulation of reactive oxygen species (ROS) during cellular activity and in response to proteins, fats, and DNA, ultimately leading to disruption of cellular processes [9]. In addition, IBP interferes with the synthesis of glucose and albumin in the liver. In addition, IBP disrupts the potential of the antioxidant system and increases free radicals and cell damage [10].

Natural sources continue to be the primary source for obtaining antioxidant molecules that can be used to prevent damage and toxicity caused by oxidation, despite the discovery of new synthetic compounds with antioxidant effects. Therefore, much research has been performed on natural products, especially those rich in polyphenols and flavonoids which have interesting antioxidant biological properties [11,12]. 

Propolis is one of these antioxidant products [13]. Propolis is a sticky, resinous substance from various plants secreted by honeybees [14]. It is a series of resinous, sticky, gummy, and balsamic substances of various compositions, collected by bees from certain plant parts (mainly the buds of certain trees) [15]. Its extracts contain large amounts of polyphenols, flavonoids, and ascorbic acid. Thus, it has strong antioxidant properties [16,17]. Many studies have shown that propolis has several biological functions such as anti-allergic activity, analgesic–anesthetic activity, antibacterial activity, immune modulatory activity, anti-inflammatory and antiviral activity, and hepatoprotective activity [14,18,19,20,21,22].

In addition, chitosan, an important polysaccharide of marine origin, is another one of these antioxidant products and it is prepared from crustaceans’ shells. It has attracted much attention as a biomedical material, owing to its anti-tumor [23], anti-ulcer, immune-stimulatory [24], and anti-bacterial activities [25]. Recently, chitosan antioxidant activity has attracted the most attention. Studies show that chitosan has hepatoprotective effects related to antioxidant activity [26]. Chitosan, a deacetylated chitin product, is a natural polysaccharide with good potential to replace synthetic stabilizers. Chitosan consists of glucosamine and N-acetylglucosamine units linked by β-1,4-glucosidic bonds. Chitosan has unique polycationic, chelating, and membrane-forming properties because it is an oxygen-rich linear polysaccharide with active amino and hydroxyl groups [27]. Therefore, chitosan has several interesting biological effects, such as biocompatibility, biodegradability, non-antigenicity, non-toxicity, and adsorption properties [23].

In addition, recent developments in nanotechnology have had a significant impact on the development and application of nanomedicine in the diagnosis and treatment of liver injury. Nanostructures with well-designed targeting capabilities and diagnostic capabilities for liver fibrosis can be used therapeutically, as contrast agents, as contrast enhancers, or as diagnostic nanoprobes [28].

Based on the antioxidant and hepatoprotective effects of propolis and chitosan nanoparticles as natural agents, the aim of the current study is to investigate the therapeutic effect of them on ibuprofen-induced hepatotoxicity.

## 2. Materials and Method

### 2.1. Preparation of the Extract

A total of 10 g of propolis dry powder extract from a local supplier (Imtenan, Cairo, Egypt) was dissolved in 1 L absolute ethanol and incubated overnight at 100 rpm. The ethanolic extract was filtered using filter paper no.1, and then the filtrate was dried using a rotary evaporator at 40 °C and 100 rpm. The yield was 8.3 g of the ethanolic extract.

### 2.2. Preparation of Propolis Suspension

A total of 1.15 mL of Tween 80 was mixed with 345 mg of ethanolic extract using a magnetic stirrer at 300 rpm. A total of 113.85 mL of distilled water was added gradually to the mixture and stirred for 30 min. The final suspension was then sonicated for 15 min.

### 2.3. Preparation of Chitosan–Propolis Nanoparticles

A stock of 92 mL of chitosan with a concentration of 0.2% (*w*/*v*) was dissolved with 1% acidic acid using a magnetic stirrer at 600 rpm. A total of 345 mg of the ethanolic extract was dissolved in 23 mL of absolute ethanol using a magnetic stirrer at 400 rpm. After complete dissolution, the two stocks were mixed at 600 rpm for 30 min at 25 °C. A stock of 0.15% (*w*/*v*) sodium tripolyphosphate (TPP) was prepared, and then 23 mL was added dropwise to the chitosan-extract mixture during stirring at 600 rpm. After adding TPP, the final formula was mixed at 600 rpm for 30 min at 37 °C [29]. The final concentration of propolis was 3 mg/mL.

### 2.4. Preparation of Chitosan-Blank Nanoparticles

A stock of 92 mL of chitosan with a concentration of 0.2% (*w*/*v*) was dissolved with 1% acidic acid using a magnetic stirrer at 600 rpm. A stock of 23 mL of blank absolute ethanol was used. After complete dissolution, the two stocks were mixed at 600 rpm for 30 min at 25 °C. A stock of 0.15% (*w*/*v*) sodium tripolyphosphate (TPP) was prepared and then 23 mL was added dropwise to the chitosan-extract mixture during stirring at 600 rpm. After adding TPP, the final formula was mixed at 600 rpm for 30 min at 37 °C [29].

### 2.5. Measurement of Particle Size and Zeta Potential

Using a particle size analyzer Dynamic Light Scattering (DLS) (Zetasizer Nano ZN, Malvern Panalytical Ltd., Worcestershire, UK) at a fixed angle of 173° at 25 °C, photon correlation spectroscopy was used to determine the mean particle size and size distribution of the prepared particles in terms of average diameters and polydispersity index. The samples underwent triplicate analysis. Zeta potential was calculated using the same apparatus.

### 2.6. Animals and Experimental Design

In the current study, thirty (30) male Wistar albino rats, weighing 90 ± 5 g were used. Rats were acquired from the animal house of the National Research Center in Cairo, Egypt. Individual rats were kept in climate-controlled stainless-steel cages in an animal care facility with 12 h light/dark cycles, a room temperature of 25 ± 5 °C, and a relative humidity of 50 ± 10%. Rats were provided with a pre-experimentation period of seven days to adapt to the lab environment. Rats were fed a regular commercial pellet diet according to the National Research Center in Cairo, Egypt (1995). Water was available at all times during the experiment. Rats were divided into 5 groups with 6 rats in each. Every other day, rats were weighed, and the doses of ibuprofen, propolis, and chitosan were modified correspondingly. Group 1 (G1), the control group, received saline solution. Group 2 (G2), ibuprofen-induced hepatotoxicity; ibuprofen was administered orally at a dose of 400 mg/kg body weight using a stomach tube. Group 3 (G3), treated with propolis; propolis was taken orally at a dose of 200 mg/kg body weight twice/week using a stomach tube [30]. Group 4 (G4), treated with chitosan; chitosan was administered orally with a dose of 200 mg/1 kg body weight/day [31] and Group 5 (G5), treated with propolis and chitosan together.

### 2.7. Biochemical Analysis

Reduced glutathione (GSH) activity, superoxide dismutase (SOD), nitric oxide (NO), and malondialdehyde (MDA) were detected in liver tissue homogenate using (Bio-diagnostic kits, Cairo, Egypt) and measured by a colorimetric method. Serum aspartate aminotransferase (AST), alanine aminotransferase (ALT), alkaline phosphatase (ALP), and gamma-glutamine transaminase activities were quantified according to the kits of (Bio-diagnostic Company, Cairo, Egypt). Serum total bilirubin and direct bilirubin were analyzed using kits which were purchased from (Bio-diagnostic Company, Egypt). Serum albumin, total protein, and globulin determination was performed by an enzymatic colorimetric method using kits from (Bio-diagnostic Company, Egypt). Serum uric acid and creatinine levels were determined by an enzymatic colorimetric method using kits developed by (Bio-diagnostic Company, Egypt). Serum urea level measurement was performed by an enzymatic colorimetric method according to [32] using kits from (Bio-diagnostic Company, Egypt). Serum phosphorous, sodium, and potassium levels were measured by an enzymatic colorimetric method using kits from (Bio-diagnostic Company, Egypt). Interleukins (10, 6, 1β), nuclear factor kappa-light-chain-enhancer of activated B cells (NF-κB), and anti-apoptotic protein (BCL-2) levels were determined in the blood using ELISA technique; kits developed by (Elabscience Company, Houston, TX, USA).

### 2.8. Statistical Analysis

Results are presented as mean ± SD. One-way analysis of variance (ANOVA) at *p* < 0.05 and least significant difference (L.S.D) were used in the statistical analysis of data via the statistical package for social science (SPSS) version 17.0 in addition to Duncan’s test to compare all groups.

## 3. Results

### 3.1. Characterization of Chitosan–Propolis Nanoparticles

Nanoparticle size reduction is known to improve the effectiveness, solubility, and bioavailability of active medicinal ingredients in a formulation [33]. The polydispersity index is an indicator of the size distribution of nanoparticles. Our formulation showed a polydispersity index of 0.236, suggesting that it is monodispersed. Because of the cationic characteristics of the chitosan molecule, chitosan nanoparticles have a positive zeta potential. Chitosan’s positive zeta potential improves medication delivery by enhancing adhesion to the negatively charged cell membrane [34]. The electrostatic repulsion between the particles rises as the magnitude of the zeta potential increases, resulting in a more stable colloidal dispersion. Our formulation was found to have a positive zeta potential of +43.0 mV as found in Table 1. Based on all of the aforementioned factors, our formulation was determined to be excellent, with an average particle size of 699.1 nm, polydispersity index of 0.236, and zeta potential of 43.0, and it was chosen for investigation of its anti-inflammatory and anti-oxidative defensive capabilities.

### 3.2. Hepatic and Kidney Function

Several hepatic markers were analyzed to examine the effect of propolis and chitosan on the treatment of ibuprofen-induced hepatotoxicity. Table 2 presents these hepatic parameters. The liver function markers AST, ALT, GGT, ALP, albumin, and direct bilirubin were significantly elevated in the ibuprofen-induced group compared with the control. On the other hand, the groups of rats treated with propolis or chitosan or the combination of both (G3, G4, and G5) showed significant improvements in the hepatic status compared to G1. However, no significant change in kidney function between all groups compared with the control (G1), and also no significant change in total protein and total bilirubin was observed. Additionally, there were significant changes in the electrolyte level (phosphorus, sodium, and potassium) when compared with the control group.

### 3.3. Oxidative Stress Status

Table 3 illustrates the levels of parameters and enzymes related to the state of oxidative stress in liver tissues of different groups. These results show that ibuprofen (G2) decreased the activity of antioxidant enzymes (SOD, GSH, and GST) in the liver tissues. In addition, the lipid peroxidation level (MDA) and nitric oxide (NO) in the liver were significantly increased in the group that received only ibuprofen (G2) compared with the control group. G4 show significant changes in oxidative parameters compared to the control group. The rats in G3 (treated with propolis), G4 (treated with chitosan), and G5 (treated with a combination of propolis and chitosan) showed significant improvements in hepatic antioxidant status parameters (SOD, GSH, and GST) and a highly significant decrease in MDA rate and NO level.

### 3.4. Inflammation Markers

We measured the concentration of pro-inflammatory (IL-1β and NF-kB) and anti-inflammatory parameters (IL-6 and BCl-2) of rats treated with propolis or chitosan and those suffering from toxicity caused by ibuprofen. Administration of propolis or chitosan or the combination of them, G3, G4, and G5, respectively, decreased the pro-inflammatory condition and improved the anti-inflammatory condition compared to the control group. In the G2 group, the level of IL-1β increased significantly in relation to G1 (32.94 pg/mg). In the same way, in the G2 group (Table 4), the level of IL-6 and BCl-2 in the liver (14.66 and 11.9, respectively) decreased significantly in relation to the control group (G1) (30.36 pg/mg and 121.99 ng/g, respectively). These results were reversed by treatment with propolis or chitosan or the combination of them (G3, G4, and G5) which showed significantly lower levels of the pro-inflammatory parameters and higher levels of the anti-inflammatory parameters compared to the control group G1. 

The findings demonstrated that ibuprofen alone significantly raised the level of malondialdehyde (MDA), significantly decreased the level of glutathione (GSH) in the liver, and significantly lowered the activity of the antioxidant system as indicated by hepatocyte superoxide dismutase (SOD) and glutathione-s-transferase. Also, ibuprofen caused a significant increase in alkaline phosphatase (AST, ALT, GGT, and ALP) activity as compared to the control group (*p* < 0.05), propolis, or bee pollen, administration with ibuprofen led to a significant reduction in MDA levels in liver tissue while significantly raising GSH and SOD activity levels. Significant alterations in the levels of phosphate and sodium in the serum were also noted. A significant change in serum sodium and phosphorus levels was also observed. The propolis, or bee pollen, supplementation enhanced serum sodium and phosphorus and potassium levels in serum as compared to the G1 group alone. On the other hand, no significant change in serum total protein, globulins, direct bilirubin, urea, creatinine, and uric acid levels as compared to the control group (*p* > 0.05) was recorded.

## 4. Discussion

Similar to other NSAIDs, ibuprofen is a propionic acid NSAID, and significantly inhibits the activity of cellular cyclooxygenases (Cox-1 and Cox-2) which prevents the production of prostaglandin, prostacyclin, and thromboxane derivatives, which are key mediators of inflammation and pain. Ibuprofen has antipyretic, anti-inflammatory, and analgesic properties. In the United States, ibuprofen was authorized for prescription usage in 1974 and became accessible over-the-counter in 1984 [35]. Ibuprofen inhibits both cyclooxygenases [7]. Free radical (ROS) levels and cell damage might increase as a result of an increase in ROS generation and a decrease in body antioxidant activity [36]. Hepatotoxicity is caused by ROS [37]. After a brief latency period, ibuprofen-associated induced liver injury frequently manifests as hepatocellular injury. There are documented cases of ibuprofen causing liver damage that required a liver transplant or resulted in death [38].

Our findings showed a highly significant elevation of ALT, AST, ALP, and GGT enzymatic activity in the rats of the group G2 that received an injection of ibuprofen. Elevation of liver enzyme activity in the blood may be caused by tissue damage in the liver, membrane permeability changes, or increased synthesis or decreased aminotransferase catabolism [39]. AST, ALT, and ALP are the main enzymes used to evaluate liver function status [40]. These enzymes are the most sensitive markers as they are directly involved in the cellular injury and toxicity degree, as they are cytoplasmic and released into the circulatory system after cell injury [41]. All of these are indications for hepatotoxicity events. 

The ibuprofen-induced liver toxicity mechanism is not completely understood but may be multi-factorial. One of these factors may be hypersensitivity responses to a toxic metabolic byproduct that accompany the liver injury and point to an allergic immune reaction [35].

Moreover, our study showed a highly significant increase in hepatic tissue MDA and NO and a highly significant decrease in GSH, GST, and SOD in the rats of the group G2 (ibuprofen-induced hepatotoxicity), which agrees with the liver metabolism of ibuprofen causing an elevation of the free radicals (ROS) by reducing antioxidants [10]. ROS can react with biomolecules and lead to molecular oxidation, i.e., lead to the elevation of NO and MDA as markers of oxidative stress diagnosis [42] and decrease in antioxidant system enzymes. 

In addition, inflammation and increased oxidative stress play a major role in ibuprofen hepatotoxicity, resulting in the necrosis and apoptosis of hepatocytes [43,44]. Necrotic hepatocytes produce damage-associated molecular patterns and stimulate the innate immune system by releasing excessive inflammatory mediators, such as nuclear factor-κB (NF-κB) and interleukin 1 beta (IL-1β), which eventually leads to severe liver injury [45,46,47]. This can explain our results showing a highly significant increase in the pro-inflammatory cytokines and mediators (IL-1β and NF-κB) and highly significant decrease in the anti-inflammatory parameters’ levels (IL-6 and BCl-2) in rats in G2 (ibuprofen-induced hepatotoxicity). 

Natural medicine is now gaining popularity around the world due to its ease, safety, and effectiveness and natural medicine refers to the practice of administering natural substances and their derivatives for the treatment of human diseases. Natural medicine treatment is integrative in nature and must use the most suitable therapies to meet the needs of the patient [48]. For this reason, we selected propolis and chitosan as natural substances and evaluated their effects on hepatotoxicity. 

Propolis is a natural substance generated by honeybees from the resin of various plant species. Propolis administration was found to enhance the activity of liver microsomal drug metabolism enzymes, significantly inhibit lipid peroxidation, and significantly increase glutathione levels in the liver and kidneys [49]. Oxygen free radical-mediated lipid peroxidation is a major contributor to cell membrane destruction and tissue damage caused by ibuprofen-mediated tissue damage. Elevated MDA levels demonstrated that co-administration of propolis and ibuprofen significantly decreased MDA formation in rat liver tissue. Propolis phenolic components and their antioxidant activity may be responsible for this effect [49].

Administration of propolis to rats restored these inflammatory markers and showed a significant reduction in oxidative damage caused by oxidative stress, which was considered to be a very important protective effect against hepatotoxicity [50].

Propolis extract contains flavonoids. By defending or boosting endogenous antioxidants, flavonoids can also have an antioxidant effect. SOD and CAT scavenge the free radicals that chemotherapy drugs activate. Numerous flavonoids have the ability to decrease oxidative stress by boosting endogenous antioxidant status, which shields cells from damage caused by free radicals and increases resistance to oxidative stress [51]. As per previous research, we observed a decrease in SOD, GSH, and GST activities in the liver of treated rats. However, these depletions were reversed when propolis was given to the rats at the same time. Our findings concur with these data, which indicate that oxidative stress is a mechanism underlying the toxicity of ibuprofen and that propolis may have a protective effect against this kind of oxidative stress, and our results agree with [51] and indicate that propolis extract possesses antioxidant properties [52].

When compared to a pure drug solution, nanoparticle-based drug delivery systems provide improved drug stability, therapeutic effectiveness, and penetrating power [53,54]. Enzymatic degradation is avoided when drugs are enclosed in nanostructured carriers with the appropriate size and surface charges [55]. Although most nanoparticle systems used to treat biofilms involve metals or pharmaceuticals, nanoformulations including natural ingredients have a greater therapeutic potential. Propolis is a natural anti-inflammatory substance that is appropriate for the development of nanoparticles.

Rats treated with chitosan showed normalization of plasma AST and ALT activities, suggesting that chitosan may stabilize the cell membrane and stop intracellular enzyme leakage into the blood [26]. Ref. [56] found that chitosan nanoparticle treatment caused this effect due to chitosan’s ability to decrease oxidative stress and improve endogenous antioxidant defenses. The decrease in liver markers observed with chitosan may be partly due to antioxidant compounds that help to reduce liver injury [23]. 

Chitosan may bind to cell membranes and cause some biological modification in the cell membrane due to its highly positively charged structure [57]. The present findings indicate that chitosan does not cause lipid peroxidation. Therefore, chitosan could interact with the cell membrane to decrease liver enzyme levels. Treatment with chitosan also has the potential to reduce the risk of liver damage [58].

Additionally, other previous studies stated that the damage caused by ibuprofen’s toxic effects is because ROS production results in damage to the various membrane components of the cell which leads to cytoplasmic enzyme infiltration [59]. Amelioration of the toxic effect of ibuprofen on liver enzymes appears when chitosan nanoparticles and chitosan with propolis are co-administered with ibuprofen, and this may be due to the ability of chitosan to stabilize the cell membrane and avoid infiltration of intracellular enzymes into the blood. Administration of chitosan nanoparticles could markedly inhibit the serum level increase in liver enzymes, which agrees with [60,61].

All of the above explained and confirmed our study results which showed improvement in liver enzymes, antioxidant enzymes, and inflammation condition in G3, G4, and G5 (treated with propolis or chitosan or a combination of them) compared with G1 (control group). 

## 5. Conclusions

Based on the results obtained, it can be concluded that natural antioxidant supplementation, such as propolis or chitosan or the combination of them, during ibuprofen administration enhanced the reduction in the toxic effects and improved both the antioxidant system and anti-inflammatory condition, as well as the levels of minerals in the serum. Observations also suggest that propolis and chitosan nanoparticles, as potent natural agents, have a hepatoprotective activity and may be effective as antioxidants in hepatotoxicity.

## Figures and Tables

**Table 1:** Physical characterization of the nanoparticle formulations.

Formulation	Average Particle Size (nm)	Zeta Potential (mV)	Polydispersity Index (PDI)
Chitosan-blank nanoparticles	774.3 ± 89.88	35.2 ± 0.874	0.438 ± 0.01
Chitosan–propolis nanoparticles	699.1 ± 75.67	43.0 ± 1.07	0.236 ± 0.01

**Table 2:** Hepatic and kidney function parameters in different groups of albino rats (G1, G2, G3, G4, and G5).

Group No.	G1	G2	G3	G4	G5	*p*
Parameter	Mean ± SE					
ALT (U/L)	27.78 ± 0.57 ^a^	44.75 ± 0.79 ^c^	34.01 ± 0.25 ^b^	33.29 ± 0.99 ^b^	32.81 ± 0.97 ^b^	<0.001
AST (U/L)	43.67 ± 1.5 ^b^	60.73 ± 1.52 ^c^	43.68 ± 1.18 ^c^	43.60 ± 1.02 ^a^	37.92 ± 1.02 ^ab^	<0.001
ALP (U/L)	184.64 ± 1.54 ^c^	283.73 ± 0.48 ^e^	180.00 ± 1.90 ^b^	191.91 ± 1.05 ^a^	149.00 ± 2.25 ^d^	<0.001
Albumin (mg/mL)	4.53 ± 0.06 ^b^	3.63 ± 0.11 ^a^	4.52 ± 0.10 ^b^	4.50 ± 0.24 ^b^	4.56 ± 0.23 ^b^	<0.002
GGT (U/L)	1.08 ± 0.04 ^c^	3.75 ± 0.18 ^d^	0.52 ± 0.04 ^a^	0.80 ± 0.04 ^a^	0.65 ± 0.02 ^b^	<0.001
T. bil (mg/dL)	0.12 ± 0.02 ^a^	0.38 ± 0.04 ^b^	0.33 ± 0.06 ^b^	0.33 ± 0.03 ^b^	0.35 ± 0.02 ^b^	<0.001
D. bil (mg/dL)	0.015 ± 0.002 ^a^	0.025 ± 0.002 ^b^	0.015 ± 0.002 ^a^	0.016 ± 0.002 ^a^	0.013 ± 0.002 ^a^	<0.001
Total protein (mg/dL)	6.52 ± 0.14 ^cd^	4.58 ± 0.06 ^a^	5.99 ± 0.14 ^bc^	6.85 ± 0.29 ^b^	5.86 ± 0.25 ^d^	<0.001
Globulins	1.98 ± 0.17 ^c^	0.82 ± 0.04 ^a^	1.41 ± 0.06 ^b^	2.35 ± 0.09 ^b^	1.31 ± 0.04 ^d^	<0.001
Creatinine (mg/dL)	0.47 ± 0.005 ^a^	0.65 ± 0.018 ^d^	0.58 ± 0.004 ^c^	0.54 ± 0.013 ^bc^	0.57 ± 0.005 ^b^	<0.001
BUN (mg/dL)	31.00 ± 0.37 ^c^	38.45 ± 0.68 ^d^	29.73 ± 0.23 ^bc^	24.43 ± 0.41 ^b^	28.67 ± 0.86 ^a^	<0.001
Phosphorus (mg/dL)	7.09 ± 0.19 ^a^	9.71 ± 0.20 ^c^	7.76 ± 0.09 ^b^	6.75 ± 0.08 ^b^	7.59 ± 0.13 ^a^	<0.001
Sodium (MEq/L)	124.55 ± 1.37 ^b^	109.17 ± 2.22 ^a^	129.66 ± 2.7 ^bc^	132.16 ± 2.12 ^c^	132.82 ± 1.52 ^c^	<0.001
Potassium (MEq/L)	0.98 ± 0.02 ^b^	0.64 ± 0.04 ^a^	1.43 ± 0.11 ^c^	3.40 ± 0.18 ^b^	1.10 ± 0.01 ^d^	<0.001
Uric acid (mg/dL)	0.48 ± 0.02 ^a^	0.95 ± 0.01 ^d^	0.66 ± 0.02 ^c^	0.56 ± 0.02 ^ab^	0.53 ± 0.02 ^b^	<0.001

a, b, c and d are resulted from (Statistical analysis) done in this study in addiation to Duncan test to compare all groups to show significance between all groups.

**Table 3:** Oxidative stress status parameters in different groups of albino rats (G1, G2, G3, G4, and G5).

Group No.	G1	G2	G3	G4	G5	*p*
Parameter	Mean ± SE					
GSH (mg/g)	271.43 ± 21.54 ^c^	164.7 ± 14.14 ^d^	558.97 ± 32.68 ^b^	320.63 ± 9.06 ^c^	742.26 ± 11.48 ^a^	<0.001
GST (U/mg)	45.74 ± 2.66 ^a^	25.4 ± 1.19 ^c^	45.18 ± 2.73 ^a^	37.21 ± 1.25 ^b^	43.31 ± 1.13 ^a^	<0.001
NO (µmol/L)	0.03 ± 0.004 ^d^	0.17 ± 0.008 ^a^	0.06 ± 0.008 ^c^	0.08 ± 0.004 ^c^	0.14 ± 0.012 ^b^	<0.001
SOD (U/mg)	140.15 ± 2.80 ^b^	39.33 ± 2.02 ^e^	165.52 ± 0.89 ^a^	77.92 ± 2.67 ^d^	87.7 ± 1.52 ^c^	<0.001
MDA (nmol/g)	88.03 ± 0.99 ^b^	103.55 ± 3.39 ^a^	40.98 ± 2.34 ^d^	58.74 ± 2.75 ^c^	82.72 ± 4.53 ^b^	<0.001

a, b, c and d are resulted from (Statistical analysis) done in this study in addiation to Duncan test to compare all groups to show significance between all groups.

**Table 4:** Inflammation markers in different groups of albino rats (G1, G2, G3, G4, and G5).

Group No.	G1	G2	G3	G4	G5	*p*
Parameter	Mean ± SE					
IL-6 (pg/mg)	30.36 ± 1.34 ^b^	14.66 ± 0.39 ^d^	22.98 ± 1.00 ^c^	23.13 ± 1.12 ^c^	42.3 ± 1.98 ^a^	<0.001
IL-1B (pg/mg)	24.19 ± 1.41 ^b^	32.94 ± 0.67 ^a^	4.07 ± 0.27 ^d^	12.74 ± 0.54 ^c^	12.42 ± 0.38 ^c^	<0.001
BCl-2 (ng/g)	121.99 ± 0.41 ^c^	11.9 ± 0.53 ^a^	165.74 ± 2.76 ^b^	103.39 ± 0.25 ^d^	170.8 ± 1.76 ^a^	<0.001
Nf-kb (pg/mg)	5.22 ± 0.10 ^b^	7.1 ± 0.22 ^a^	4.54 ± 0.18 ^c^	3.68 ± 0.22 ^d^	2.83 ± 0.26 ^e^	<0.001

a, b, c and d are resulted from (Statistical analysis) done in this study in addiation to Duncan test to compare all groups to show significance between all groups.

## Data Availability

The datasets collected and/or analyzed during the current study are available from the corresponding author on request [Hussein Mohamed]. The corresponding author had full access to all the data in this study and takes responsibility for the integrity of the data and the accuracy of the data analysis.
